# On Factory Labour

**Published:** 1833-02

**Authors:** John Malyn

**Affiliations:** Surgeon.


### FACTORY LABOUR.

*On Factory Labour.* By John Malyn, Esq., Surgeon. (Read before the Westminster Medical Society.)

An inquiry into the influence which labour exerts on the animal economy, when that labour is pursued, during the process of maturation, in a heated and polluted atmosphere, in an erect and slightly-varying position, and with but brief remissions for refreshment and rest, would possess considerable attraction, even as a speculative question, totally unconnected with the actual condition of beings who had been subjected to the implied experiment. It inspires, however, a deeper interest when its object is not only to demonstrate that the moral as well as physical degradation of such as are so exposed, is the natural and unavoidable consequence of their employment; but also, by fairly and dispassionately stating the grounds on which that conviction is founded, to offer such evidence of the iniquity of the system as to secure, in the design of its improvement, the co-operation even of those persons who, regarding it as a mere commercial question, have hitherto been opposed to the benevolent attempts which have been made to mitigate the abiding misery of the poor.

Great as is the evil, and widely and severely as it has been extended, even to the alteration of character, the engendering of disease, and the abridgment of the natural period of life, it is yet of comparatively recent origin.

Before the ingenuity of the mechanic had called into existence those potential instruments of manufacture which bring forth wealth in unbounded profusion, and cast into shade the former humble and unassisted operations of man, the different fabrics of the country were produced in the cottage of the artisan. Surrounded by those in whom he took a direct interest, he laboured in his vocation with advantage to himself and family: his daughters became qualified for the discharge of domestic duties; his sons assisted him in his work, to the extent of their infantile powers; and the minds of both were preserved from contamination, and imbued with those hallowed sentiments which formed the seductive character, of which we have all read, but few seen specimens, in which the chief attractions are, singleness of heart and sincerity of purpose; cleanliness and health combined; devotion from affection, not fear, for their superiors; attachment to their kind, and a modest pursuance of that moral line of rectitude from which no well-regulated mind would ever have desired to see them deviate. Then it was that infancy was passed in innocent recreation, until the system was so far developed as to justify the appropriation of its powers, not as a matter of gain, but to qualify it for its part in life. The circumstances by which the child was surrounded were salutary; its introduction to labour was gradual; its powers of endurance were never exhausted, and it therefore flourished and attained strength, like a plant in appropriate soil and genial atmosphere. Acknowledging the responsibility of a parent, a workman of those days would have spurned the idea of consuming, in its youth, the manhood of his child, or of coining into money its health, its happiness, its life.

But their condition changed. Machinery was introduced, and it brought forth abundantly. The application of steam was effected, and its mighty energies multiplied the product. The cottage was swept away, and the social hearth made desolate. Men were congregated together in large establishments, to aid the operation of the engine, of which they now became the mere instruments. It was soon discovered that, as direct strength was not required, women might be serviceable; and, accordingly, for this employment they abandoned their household labours. Untiring in the pursuit of wealth, the employer failed not to observe, that a child, nay, an infant, (and that without distinction of sex,) might supply the place of an adult; and the fields and the playgrounds ceased to resound with the noisy hilarity of youth. But the evil ended not here. Individuals, whose thirst for gold was insatiable, gradually extended the period of labour. Exaction begat exaction to such an extent, that not only did the term of exertion frightfully exceed that allotted for rest, but the intervals for refreshment were at first curtailed, and afterwards, in some instances, abrogated altogether, food being taken as opportunity might serve, while they were executing their duties. It mattered not that this abuse had a local origin: having been commenced by a few, others were constrained to follow in their wake, by the coercive influence of competition. Hence have the habits and condition of these unfortunate people undergone a revolution which it is deplorable to contemplate. Their habitations are neglected and unhealthy; their food is poor and innutritious; their persons are dirty and slightly clad; their language and conduct are of the most repulsive character; females are no longer qualified for the discharge of the duties of a wife or a mother: domestic life having lost its charms, husbands seek other society and other pleasures, by indulgence in which their minds are brutalized; the children of such parents rise hours before daylight from their wretched pallet, to encounter, with scarcely any clothing, and without any food, the frost, the snow, the rain, or the bleak winds of a winter's morning. The action and reaction of these conditions aggravate each other, and produce a state of debasement that we cannot witness without concurring in the fear of the late Sir R. Peel, "that the great effort of British ingenuity, instead of being a blessing, would prove its bitterest curse."

Some reformation was effected in the cotton-factories by this gentleman, who endeavoured, and, to a certain extent successfully, to check the premature employment of children. He obtained for their protection an Act of Parliament, by the provisions of which, children under nine years of age were deemed inadmissible, and the period of labour was fixed at twelve complete hours out of the twenty-four, independent of the time allotted for meals. The Act, however, did not extend to other descriptions of factories. In them is exhibited a melancholy specimen of the atrocity which cupidity will justify, nay, defend; and they show how completely man may forget that his less fortunate fellow-creatures partake of the same nature and are formed by the same hand as himself. Thus, for instance, it was stated in the house of Commons, by that anxious friend and powerful advocate of oppressed infancy, Mr. Sadler, that, in Wales, "the children work twenty-four hours every other day, out of which they are allowed three hours only for meals, &c.;" that, "when trade is particularly brisk, the elder children work from six in the morning to seven in the evening, two hours being allowed for meals, &c., and every other night they work all nighti:"—that, at Bradford,

"Children of these years (thirteen) are obliged to be at the factories, winter and summer, by six in the morning, and to remain there till seven in the evening, with but one brief interval of thirty minutes, every day except Saturday, ceasing work on that day, in some factories, at half-past five, in others at six or seven P.M. Not unfrequently this labour is extended till eight or nine at night, fifteen hours, having but the same interval for meals, rest, or recreation: nay, such is the steady growth of this overworking system, that children have been confined in the factory from six in the morning till eight at night, fourteen hours, continuously, without any time being allowed for meals, rest, or recreation; the meals to be taken while attending the machines; and this the practice of years."

That, in a mill at Leeds, the following was the routine of employment:

"On Monday morning, work commenced at six o'clock: at nine, half an hour for breakfast; from half-past nine till twelve, work. Dinner, one hour; from one till half-past four, work. Afternoon meal, half an hour; from five till eight, work: rest for half an hour. From half-past eight till twelve (midnight), work; an hour's rest. From one in the morning till five, work: half an hour's rest. From half-past five till nine, work: breakfast. From half-past nine till twelve, work: dinner; from one till half-past four, work. Rest half an hour; and work again from five till nine o'clock on Tuesday evening, when the labour terminated, and the gang of adult and infant slaves was dismissed for the night, after having toiled thirty-nine hours, with brief intervals (amounting to only six hours in the whole) for refreshment, but none for sleep. On Wednesday and Thursday, day-work only. From Friday morning till Saturday night, the same prolonged labour repeated, with intermissions, as on Monday, Monday night, and Tuesday; except that the labour of the last day closed at five."

With such extreme cases I shall not interfere. The facts themselves appeal forcibly to the heart. No one can fail to recognize their deadly tendency; and none, possessed of feeling, will hesitate to reprobate them.

In advancing the proposition, that "the Factory System is opposed to the healthy development of the human body, however favorable may be its external relations," I state a conclusion to which I have been led, by an observation of the persons who have been employed in manufactories, in which the legislature has already interposed; and which, I have reason to believe, are conducted with as much propriety and humanity as the present system will admit.

In these establishments, the temperature is unavoidably high, although it is not the same throughout the whole of them; the variation being caused by the different degrees of fineness of the material to be produced. Thus, while the thermometer stands in one mill at fifty-five, in a second it will be at seventy, in a third at sixty-two, and, in a fourth at eighty-five.

But, whatever may be the degree, it is necessary that it should be uniform, since the object of the manufacturer would be defeated by a current of air passing through the rooms. Hence is the atmosphere quiescent, experiencing none of those undulations, so grateful in an equally high but natural temperature. In the winter months, labour is pursued in the evening by artificial light, whereby the heat is much augmented, as well as the nature of the atmosphere changed. It is of some importance to remember this fact, as this maximum of temperature exists prior to the exposure of the work-people to the external night-air.

The medium they respire is considerably affected by the constant extrication of dust and fine particles of cotton, by which it becomes charged with foreign matter. The oil of the machinery being decomposed by the heat, and the metal wheels, in this unceasing revolution on each other, extricating a powerful and oppressive noisomeness, the purity of the air is still further impaired. It suffers yet more pollution from so many persons being assembled together, who, in the respiratory process, abstract its vital principle; and the same principle is also consumed in supporting artificial light. In both instances a deleterious product is formed, which cannot be sufficiently supplied by fresh air without diminishing the temperature or injuring the work.

There is but little actual labour, and it has, therefore, been termed a light and easy occupation, as the child has scarcely any thing to do but to remain nearly stationary on his legs, and to occupy himself in a constant succession of the same acts.

The term of labour (fourteen hours) is interrupted by the morning, mid-day, and evening meals; one hour being allowed for dinner, and half an hour for each of the other two meals. It is a common practice with many children to bring their food to the mill. This, being put aside, becomes defiled by the deposition of the dust and flue; and, on this account, as well as from languor, the child often nauseates it, and either rejects it entirely, or swallows it without mastication.

A day thus spent is followed by passing into a cold, humid atmosphere, from one which is as hot as, and far more impure, than that of the tropics.

Such are the circumstances by which they are surrounded; and it is on these circumstances that we are to reason and determine their probable influence.

A stranger, whose eye had not become familiarised to the sight, would at once be struck with the inferiority and with the general aspect of debility of these operatives. Old age is unknown to them but by the anticipation of its attributes; for their services are valueless beyond forty or fifty. Instead of the hue of health, there is a sickly sallowness, languor in the place of vigorous elasticity, and weak and flabby muscles supply that tonic development produced by employment under favorable circumstances. In complexion, in size, in stature, they stand forth a race, blighted, not so much from original defect in the germ, as from the inappropriateness of the soil in which it has been reared. The diseases which prevail among them are, for the most part, diseases of debility. Acute affections are of but rare occurrence among those individuals who have, for some time, been subjected to the influence of these agents. Scrofula, in all its gradations, from its mildest to its worst form, attacking the joints, the glands, the skin, the eyes, and the viscera, is so widely spread that the disease seems here to have found its peculiar habitat. Among those who entered on the employment at an early period, and have survived the perils of youth, there are numerous cases of deformities. Many exhibit a flatness of foot which impedes progression; others, curvature of the long bones in the different directions of their length, or lateral distortion at the ankle and knee joints. It has, further, been remarked, by those engaged in that department of practice, that women who have passed their childhood in these places very frequently require instrumental assistance in parturition. In the domain of the physician, rheumatism, chronic affections of the liver, the innumerable forms of dyspepsia, local and general effusions, bronchial disease, and phthisis, are abundantly found.

In an examination, instituted some years since, for the information of parliament, of the persons employed in a number of mills, the physician and surgeon reported that out of the number *then actually at work* not one fourth of them was free from disease. Out of 266 who were working in one mill, they found that sixty-three laboured under a cough, and that seventy-five were delicate. There were fourteen cases of distortion, eight were stunted, and there were eight cases of scrofula. Besides these, there were others with enlarged joints, difficulty of breathing, headach, rheumatism and pain in the legs. And these were found, not in an hospital, as at first sight might be imagined, but in a factory, where the people were following their daily occupation.

Dark as is this picture, I should not be surprised if, without departing from truth, it might have been more highly coloured. Such examinations are very fallacious: they present the most favorable side of the question. To render them any thing near perfect, it should be known how many have been employed within a given number of years; how many have died; and what is the present condition of those who have ceased from labour. The most correct evidence which we can obtain on this point is from authentic returns of the mortality of the agricultural and manufacturing districts. From a return made to the House of Commons in July last, we learn that there die, under twenty years, in Rutland, 37½, and in the metropolis, 46 per cent.; while, in the manufacturing districts, it ranges between 57 and 62 per cent.; demonstrating that, from some cause or other, there is here an immoderate consumption of human life.

These are the effects which, without any reasoning on the subject, must lead us to infer the deleterious influence of factory employment. Why should the grave absorb the young, and the infirmity of age replace the strength of manhood? Why should the natural term of life be abridged, and the efficiency of the subject circumscribed? Why should disease be here extensive in its progress, and odious in its character? To him who has possessed himself of an accurate knowledge of the structure and functions of the human body, of the laws by which they are regulated, of the dependence and reciprocal influence they have on each other, and of the effect of external agents upon them, the answer cannot be difficult.

The alleged causes have already been stated, and we advance, therefore, to the consideration of the mode in which they act.

Among the foremost of these causes is the high temperature, which, by prejudicing the digestion, and favoring the establishment of local congestion when cold is applied, produces many forms of disease. The cutaneous circulation is stimulated by the heat, so much so as to produce perspiration; and in this state of excitement they are thrice, daily, exposed to the agency of cold air. The disturbance of the circulation consequent on this exposure may be, and often is, attended with immediate and apparent injury; but it is no less certain that the powers of nature will repeatedly restore the balance. Yet this cannot occur often with impunity; for, however trifling and imperceptible may be its effects in each instance, they silently yet surely lay the foundation of severe and enduring disease. Among the many chronic affections which thus originate, we may trace to this cause that derangement of the liver which, to a greater or less extent, is universally obvious. It has been stated that these people are characterised by a sallowness of complexion, and it is exactly what might have been anticipated from the continuance of the biliary principle in the blood, by reason of the liver being, from its torpidity, (the result of these repeated collapses,) unequal to its extrication. A somewhat similar state of skin is observed in those who have long resided in hot climates; and it is to this cause that the condition is attributed by Dr. James Johnson, in his luminous exposition of the sympathy which exists between the capillaries of the skin and the hepatic extremities of the portal vessels. The secretion of bile being imperfect, the chyme in the duodenum is deprived of a necessary agent in chylification: the product is therefore vitiated, the nutrifying portion being inappropriate to the exigencies of the system; while the fæcal remains are, from not possessing that stimulating quality which healthy bile communicates, imperfectly evolved, and, by their detention, prove a great source of intestinal irritation. It interferes with the digestion still further. An hour for dinner is sufficient for the ordinary circumstances of domestic life: it affords time for that brief repose which is necessary to the chymification of the food. The period occupied in passing to and returning from their homes reduces that interval, and originates the baneful practice of swallowing their meals without adequate trituration. I need not remark how pernicious this custom is in itself; but when, in addition, we reflect that they resume their labour with stomachs recently and improperly loaded, another important means by which the heat acts prejudicially is at once apparent. That admirable law, by which the blood is prevented flowing from the viscera through the liver, when that fluid recedes from the surface, is not only a provision against oppression of the heart in disturbed circulation, but is intended by nature to assist the action of the stomach in its early operations, as is evident by the chilliness which as invariably succeeds a full meal as it does other cases of visceral congestion. Here, however, nature is thwarted. Heat and labour conjoined determine the blood to the cutaneous vessels, by which the hepatic collapse is consequently removed. Thus, its distention about the stomach is prevented, and that organ deprived of a powerful aid assigned to it by nature. We, therefore, find matter, imperfectly masticated, subjected to the action of a stomach of which the energy is diminished, to be converted into chyme; this chyme passing into the duodenum, and failing there to receive those healthy fluids which are essential to its conversion into chyle. As the condition of the body is according to the degree of perfection of the matter by which it is sustained, we cannot be surprised that these poor creatures are of depraved and imperfectly developed structure, or that they exhibit such numerous instances of diseases of debility.

That bronchial and pulmonic affections are prominent in the deadly list, is a natural consequence of the vicissitudes of heat, and cold, and moisture, which they have to endure; but they may be increased in number and character, by the irritation of the loaded and otherwise impure atmosphere they have to respire.

We proceed now to the important consideration of its effects on early life; a branch of the question which I commend to your especial attention, as on it you may be required, before long, to pronounce an opinion.

That early and indiscriminate employment; that the child of ten should work to the same degree as a boy of fifteen, and that the latter should be employed the same number of hours as a man, is an impolitic, cruel, and atrocious practice: that it is contrary to every principle of science, and that it is an outrage on the laws of nature, must, in my opinion, be confessed by every one who is not blinded by interest or deficient of understanding.

The nature of a child differs widely from that of an adult: it appears to revolve in a more limited orbit, and, from this limitation, to be subject to more brief but rapid and quickly-recurring sensations, owing to the excitability which obtains in youth being soon exhausted, and requiring long periods of repose for its reproduction. The demand for sleep and varied occupation, whether of mind or body, is great and frequent. There is a physiological excitement of all the functions (great in proportion to the youth of the subject,) which renders them peculiarly sensitive to external impressions: this excitement is manifested by a quick transition from one thing to another, either in speaking, working, or playing; by a rapid pulse, quick respiration, profuse perspiration, and copious secretion of urine. And is it in a state like this, one so predisposed to disease, that we would consign a child to those influences under which the matured frame must eventually succumb? Further, dependent on this excitement (by which is caused a great expenditure of power and matter, from the activity of interstitial absorption,) there is the necessity for a frequent exhibition of food, not more to supply that loss than to provide for, and indeed to effect, the increase of the body. But it has already been seen that the regulations of factories will not admit this, and the development of the frame is thereby retarded, as well as the constitution weakened, by the privation. Granting that it is sufficiently often administered, its assimilation cannot be effected without good air and judicious exercise; and it has been shown that, even in the adult, in whom the functions are more steady and equable, this essential process cannot be accomplished.

To qualify a being for endurance in the active period of life, it is requisite that he acquire tone and energy, which can only be obtained by a varied action of the muscles during childhood, in a healthy atmosphere, and by at all times stopping short of fatigue. This vigour depends on the degree to which the blood is arterialized. Thus, we find that birds, whose structure is extensively permeated by air, possess a muscular force totally at variance with their size, enabling them to defy the height of mountains and the expanse of seas. The inactivity and unwholesomeness of a factory life are directly opposed to the attainment of this physical excellence, the real wealth of the labourer, and have a direct tendency to produce debility. The blood in the extremities, being deprived of the propulsive aid of the muscular system, lingers in its vessels, instead of being returned with regularity to the heart, producing, besides its general effects, local loss of tone, which, in after life, will be shown by œdematous legs, indolent ulcers, and varicose veins. The want of this tone in the muscles disqualifies them to preserve the different parts of the body, the one upon the other, for any length of time. Under the most salutary treatment, it would not be possible for youth to preserve the erect posture, especially if stationary, more than a short time, without severe suffering: it may not be pain, but it is a weariness, agonizing beyond expression, which attends muscular action when carried beyond its limits; it deadens all the energies, and weakens all the functions. Can any one reflect on the weight of the head and trunk, the number and position of the joints, and the consequently great muscular force which must be employed to preserve all these parts erect, and not shudder at the idea of being condemned to stand, like these youths, during twelve out of the twenty-four hours? The deformities which have been mentioned are readily explained, and the cause is so evident that it is almost needless to dwell on it. We well know how incomplete are the bones of the young, and that the deposition of the earthy matter, which constitutes their firmness, is not completed until a considerable time after the age of puberty. All are acquainted with the flexibility of these organs while the animal matter predominates, and it is probable that most have witnessed cases in which the application of force has bent the long bones into the form of a bow, without their being broken. At the period of life of which we are speaking, the bones are quite incompetent to the support of the superincumbent weight; by which we learn that nature (who has provided for every being, according to its condition, adequate instruments wherewith to fulfil its duty,) never designed that these bones should be submitted to such a task. Being, however, so submitted, they, from their pliability, yield, and a curvature, in one or other of the aspects of the bone, will be the result. The direction will be determined by the preponderance of power in some of the muscles, the unequal action of which in a state of fatigue principally produces the angular distortion at the joint; a facility for their operation being, perhaps, afforded, though that is problematical, by a relaxation of the lateral ligaments of the joint. The lassitude and atony of body resulting from long standing will induce the young sufferer to seek relief by some contortion of his body, throwing himself into a position in which he experiences temporary ease. He thus takes the burden from one set of muscles, to impose it on another; and, by this habit, the former acquire a certain degree of energy: the antagonising muscles, being proportionally debilitated, are unable to counteract the superior power of the other; these, then, not being sufficiently counterbalanced, they will pull the lower part of the limb against the upper in their direction, and ultimately establish its eversion or inversion, according as the influencing muscles are to the outer or the inner side. The weight of the whole body being transmitted to the astragalus, the pressure of that portion of the bone which is on the inner plantar ligament effects the relaxation of that ligament, the bones descend, and the arch of the foot (that beautiful mechanism by which elasticity of movement is secured,) is destroyed, and that flatness of foot, which is reported to be common among them, is produced. The female child incurs a greater danger: the pelvis, not being ossified, will give way to the pressure which itself transmits from above to the thighbones. This, of course, must narrow its diameter in the direction of the pressure, causing the evolution of the fœtus, in subsequent life, to be impeded, if not prevented; and thus rendering necessary that revolting operation in which one life at least is destroyed, and the other placed in imminent peril.

By a careful perusal of the book of nature, we find there is an ample provision for the duties of every state, and that those duties may be inferred from the degree of perfection which the various organs have attained. It teaches us that as, from infancy to beyond puberty, nature is occupied in progressively adding to and completing the body and the mind, so she intended that exertion of the respective organs of either one or the other should be graduated and apportioned to the extent to which she has carried her work. That, in fact, it is an initiatory state, in which the mental and physical organs are to be progressively *taught* those duties they will have actively to execute, when she herself shall have finished her design.

I cannot resist the impression that many of the mill-owners have sanctioned the abuse and opposed its redress from mere thoughtlessness, and ignorance of its consequences. They established the custom without asking themselves if it were just, and interest has since prevented them from making the inquiry. I entertain a lively hope that, so soon as they are made sensible of the desolating effects of the system, and of the incapacity of the growing frame to withstand them, even they will acknowledge that wealth and power are acquired at too high a price by the introduction and perpetuation of deformity and disease, by the abridgment of the utility of man, and by the inordinate sacrifice of life. There are among them men of high character, who profess (and I doubt not for a moment, sincerely) to feel acutely the miseries of the labourers in the plantations of the West; and who manfully advocate the restitution of what they deem their rights. They have made eloquent appeals in behalf of those they have never seen, and of whose condition they are entirely ignorant. I blame them not; but, while they are so alive to the oppression of the well-fed negro, whose hours of daily labour are limited to nine, and the services of whose children a planter cannot appropriate before the age of fourteen, it is deeply to be regretted they were ignorant that at home there were thousands upon thousands, in favor of whom their benevolence might be judiciously exercised, and whose oppression far exceeded the alleged thraldom of the slave. See p. 501.

*Duke street, Westminster; Nov.* 1832.

